# Anther Morphological Development and Stage Determination in *Triticum aestivum*

**DOI:** 10.3389/fpls.2018.00228

**Published:** 2018-02-23

**Authors:** Richard G. Browne, Sylvana Iacuone, Song F. Li, Rudy Dolferus, Roger W. Parish

**Affiliations:** ^1^Department of Animal, Plant and Soil Sciences, AgriBio, Centre for Agribioscience, La Trobe University, Bundoora, VIC, Australia; ^2^Melbourne Polytechnic, Epping, VIC, Australia; ^3^Agriculture and Food, Commonwealth Scientific and Industrial Research Organisation, Canberra, ACT, Australia

**Keywords:** anther, apoptosis-like PCD, developmental staging, *MYB80*, tapetum, *Triticum aestivum*

## Abstract

Anther development progresses through 15 distinct developmental stages in wheat, and accurate determination of anther developmental stages is essential in anther and pollen studies. A detailed outline of the development of the wheat anther through its entire developmental program, including the 15 distinct morphological stages, is presented. In bread wheat (*Triticum aestivum*), anther developmental stages were correlated with five measurements, namely auricle distance, spike length, spikelet length, anther length and anther width. Spike length and auricle distance were shown to be suitable for rapid anther staging within cultivars. Anther length is an accurate measurement in determining anther stages and may be applicable for use between cultivars. Tapetal Programmed Cell Death (PCD) in wheat begins between late tetrad stage (stage 8) and the early young microspore stage (stage 9) of anther development. Tapetal PCD continues until the vacuolate pollen stage (stage 11), at which point the tapetum fully degrades. The timing of tapetal PCD initiation is slightly delayed compared to that in rice, but is two stages earlier than in the model dicot Arabidopsis. The *MYB80* gene, which encodes a transcription factor regulating the timing of tapetal PCD, reaches its peak expression at the onset of tapetal PCD in wheat.

## Introduction

Anther development and pollen production are particularly sensitive to abiotic stresses such as heat, leading to severe reductions in crop yield (Hedhly et al., 2009). The sensitivity of the anther to abiotic stresses varies throughout the development of the anther (Saini and Westgate, 2000; Dolferus et al., 2011). An understanding of the effects of these stresses on pollen and anther development requires an accurate and efficient method to identify the various anther developmental stages.

Morphological developmental staging schemes have been previously published for Arabidopsis (Sanders et al., 1999) and rice (Raghavan, 1988; Zhang and Wilson, 2009). A 10 stage model of anther development has also been described for the model monocot *Brachypodium distachyon* (Sharma et al., 2015) and several wheat stages (although not a complete developmental program) have also been identified (Mizelle et al., 1989). Zadoks et al. (1974) and Large (1954) provide general descriptions of plant development and the Zadoks Scale has been adapted to correlate node number and flag leaf elongation with spike length in barley (Gómez and Wilson, 2012). Waddington et al. (1983) developed a scoring system for both barley and wheat which correlated spike development with anther length, spike length, awn length, and spikelet number. Floret size and anther length have been correlated with stages of anther development in rice (Raghavan, 1988). Generally, anther and pollen stages appear to be tightly linked to spike size in barley and wheat (Waddington et al., 1983; Kirby, 1988).

Auricle distance is the plant measurement commonly used in the study of anther development. The distance between the auricles of the flag and penultimate leaves of the tiller has been used to predict the onset of anther meiosis in wheat (Morgan, 1980; Ji et al., 2010) and rice (Satake and Hayase, 1974; Heenan, 1984; Oliver et al., 2005). Auricle distance has been employed to determine the development of the anther for the application of abiotic stresses including drought (Ji et al., 2010; Onyemaobi et al., 2017) and cold (Oliver et al., 2005; Dolferus et al., 2013) in wheat, as well as heat stress in rice (Endo et al., 2009).

In this paper, we determine the cellular changes occurring during development of the wheat anther. We identify and categorize these developmental stages and observe the ability of five plant tissue measurements to accurately predict anther development stage in several wheat cultivars. The findings of this study provide a simple and effective means of identifying wheat anther developmental stages.

## Materials and methods

### Plant material

Wild-type wheat seeds (*Triticum aestivum*, cv. Halberd, Cranbrook, Young, and Wyalkatchem) were obtained from CSIRO Agriculture & Food, Canberra, Australia. Plants were grown in a glasshouse at 22°/15°C, on a 14 h day/10 h night light cycle. Plants were grown in black, 220 mm diameter pots containing potting mix, vermiculite and perlite. Each pot contained four plants. A minimum light intensity of 500 μmol m^−2^ s^−1^ during the day was achieved using supplemental lighting.

### Measurement of indicators for anther developmental stages

**Figure 1:**
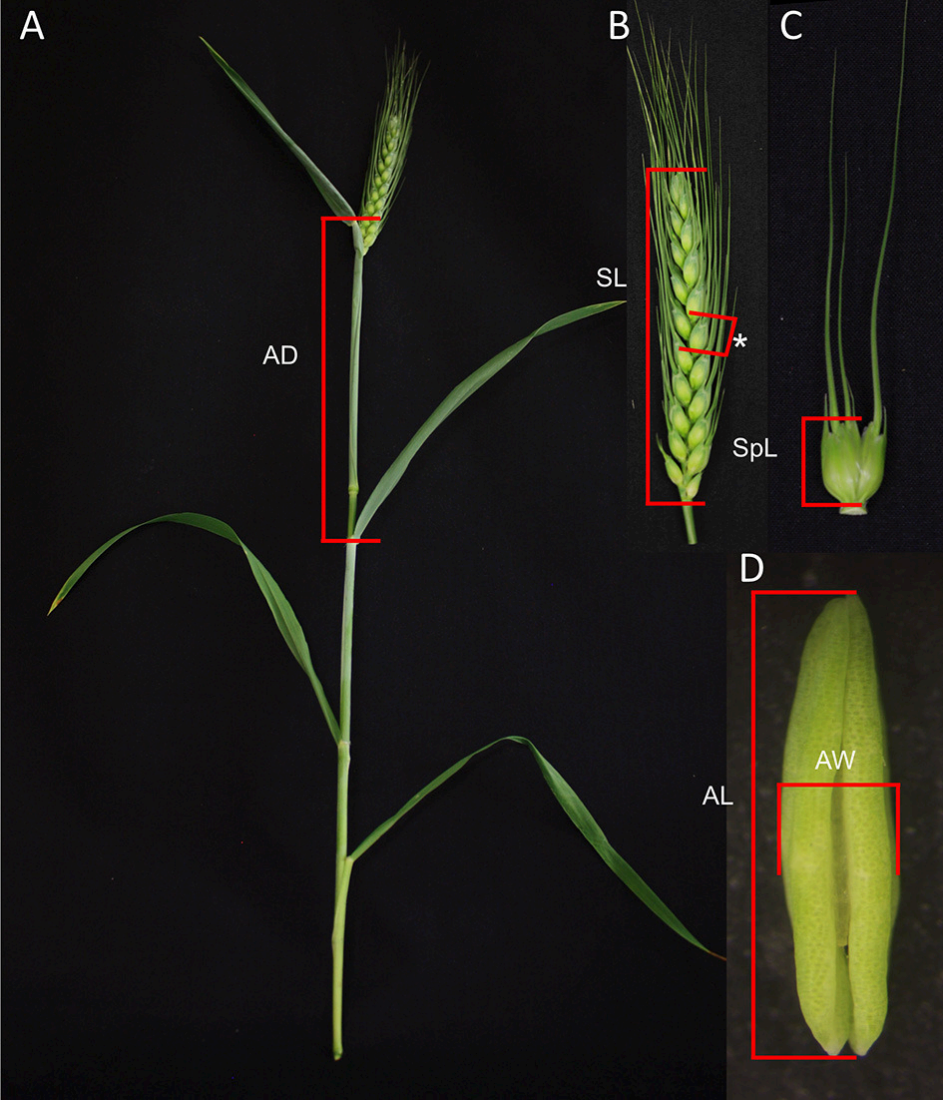
Plant measurements utilized in predicting anther developmental stage in wheat. **(A)** Auricle distance (AD) identified on a wheat tiller. **(B)** Spike length (SL) indicated on a wheat spike with a central spikelet indicated with an asterisk. **(C)** Spikelet length (SpL) indicated on a spikelet. **(D)** Anther length (AL) and anther width (AW) identified against an anther.

For all measurements, only the main stem and largest primary tillers of each plant were utilized. Auricle distance of these tillers was measured as the distance between the auricles of the flag leaf and the penultimate leaf (Figure 1A). Spikes were measured from the attachment of the lowest spikelet to the pedicel/rachis and up to the tip of the top spikelet but excluding the awns (Figure 1B). To determine spikelet length the largest spikelet at the center of the spike was selected and removed from the spike before being measured. The spikelet was measured from the base, i.e., where the spikelet attaches to the rachis, to the tip of the central floret, excluding any awns (Figure 1C). Anther length was determined following removal of the anther from the primary floret of the largest spikelet (Figure 1D). Anther width was the width of the anther at its broadest point (Figure 1D). Spikelet and anther measurements were performed using a calibrated graticule eye piece in a Wild Heerbrugg M38 dissecting microscope. Auricle and spike measurements were carried out manually. Auricle distances were only recorded when both auricles were visible.

### Resin sectioning of anthers

Anthers at varying stages of development were fixed in 6% glutaraldehyde and 4% paraformaldehyde in 50 mM sodium phosphate buffer (pH 7.5) for 2 h at 4°C followed by 15 min of vacuum infiltration (Li et al., 2007). Fixed tissue was dehydrated using the following ethanol gradient series: 30, 60, 70, 90, and 100%. Subsequently, samples were infiltrated with LR white resin/ethanol. Anthers were embedded overnight at 65°C and sectioned using a Reichert ultramicrotome (Reichert, Depew, NY, USA). Semi-thin transverse sections (3.0 μm) through the center of the anther were stained with 1% toluidine blue. Sections were viewed with brightfield conditions using an Olympus BX53 Upright Microscope, captured with the Olympus cellSens Dimensions software, and photographed with an Olympus DP80 Color microscope camera (Olympus, Shijuku, Tokyo, Japan).

### Paraffin wax embedding of anthers

The largest two or three spikelets were dissected from the spike and the less mature inner florets were removed, leaving only the primary and secondary florets of each spikelet. Spikelets were fixed in 4% paraformaldehyde in a PBS solution for 30 min under vacuum and incubated for 2 h at room temperature. Vacuum infiltration and room temperature incubation were repeated once. Samples were washed with PBS and dehydrated using an ethanol gradient (50% twice, 60, 70, 85, and 95%) for 30 min in each solution. Samples were left overnight in 95% ethanol including 0.1% eosin and then washed twice for 1 h in 100% ethanol. Samples were cleared in xylenes/ethanol solutions of increasing concentration (1:2, 1:1, 2:1) for 30 min each. Samples were incubated twice in 100% xylenes for 1 h. Spikelets were placed in molten paraffin wax, which was changed five times, before being embedded in paraffin wax. This method is according to Phan et al. (2011).

### Tunel assay

Wax embedded samples were sectioned to 6 μm using a Jung rotary microtome (Reichert, Depew, NY, USA) and attached to a silane-coated slide. Samples were deparaffinized in 100% xylenes and dehydrated in a graded ethanol wash (100, 95, 85, 70, and 50%) for 3 min each. Samples were washed in PBS and 4% paraformaldehyde prior to being permeabilized in a 20 μg/ml Proteinase K solution for 4–5 min. Nick-end labeling of the fragmented DNA was performed using the DeadEnd™ Fluorometric TUNEL System (Promega, Madison, WI, USA) according to the manufacturer's protocols. Slides were stained in a 1 μg/ml propidium iodide solution with PBS for 15 min and mounted with SlowFade® Gold Antifade Reagent (Invitrogen, Carlsbad, CA, USA), before a cover slip was sealed using clear nail varnish, with samples stored at 4°C in the dark. Confocal microscopy was performed using a Leica TCS SP2 Fluorescence Scanning Confocal Microscope (Leica microsystems, Wetzlar, Germany), employing excitation/emission of 488/509 nm to view fluorescein, and excitation/emission of 538/617 nm to view the propidium iodide. Images were merged using Image J (National Institute of Health, Maryland, USA).

### Quantitative real time PCR analysis

Anther stages were determined using anther length measurements, and anthers of different stages were separated into five developmental pools which were used for qRT-PCR analyses. For each stage, four biological replicates were collected, comprising approximately 100 anthers taken from four individual spikes. Anthers were collected from the primary and secondary florets of the largest four or five spikelets of each spike. Once dissected, anthers were immediately placed in RNAlater® (Ambion, Thermo Fisher Scientific, Waltham, MA, USA). After collection, RNAlater was removed and anthers were frozen in liquid nitrogen and stored at −80°C until RNA extraction was performed. RNA was extracted using the Isolate II RNA Plant Kit (Bioline, Sydney, Australia) according to the manufacturer's protocol ‘Purifying total RNA from plant tissue or filamentous fungi’. RNA samples were checked for concentration and purity using a NanoDrop ND-1000 (Thermo Fisher Scientific, Waltham, MA, USA) and stored at −80°C.

DNA contamination was removed using DNase (Deoxyribonuclease I, Amplification Grade) (Invitrogen, Carlsbad, CA, USA). RNA to cDNA synthesis was performed using SuperScript™ III Reverse Transcriptase (Invitrogen, Carlsbad, CA, USA), according to the manufacturer's protocols. The PCR conditions for the cDNA synthesis were 50°C for 45 min followed by 70°C for 15 min. The cDNA samples were confirmed to be DNA free using PCR amplification. Primers and conditions were optimized using standard curves so that a single melt curve was produced and primers met the following conditions: *E* = 90–110%, *R*^2^ ≥ 0.985, slope = −3.1 – −3.6. The conditions for qRT-PCR were as follows: 95°C for 2 m; 40 cycles of 95°C for 20 s, 60°C for 30 s; 95°C for 15 s; 60°C for 1 m; melt curve up to 95°C. The qRT-PCRs were performed using SensiFast™ SYBR and Fluorescein Mix (Bioline, Sydney, Australia) with a QuantStudio 12K Flex (Applied Biosystems, Life Technologies, USA). Two genes, an ADP-ribosylation factor (Ta2291) and a Cell Division Control protein, AAA-superfamily of ATPases (Ta54227) were utilized as reference genes. Primers for the reference genes used in this study were selected from previous publications (Paolacci et al., 2009; Nancarrow et al., 2014). The geometric mean of the reference genes was utilized for gene expression normalization. Averages of four technical replicates were used to determine individual Ct-values for genes of each biological replicate. The ΔCt for each biological replicate was averaged, and relative transcript abundance was calculated as 2^−ΔCt^ compared to the geometric mean of the reference genes. Error bars represent standard error. All primers used are listed in the Supplemental Table 1.

### RNAseq expression

#### Sampling for transcriptomics

Plants were grown in the glasshouse for approximately 6–7 weeks. Auricle distance was utilized to predict the developmental stage of anthers. Tillers in which the anthers of the largest spikelets were determined to be at either the meiotic or tetrad stages of development were tagged and at the beginning of the day cycle plants were moved from the glasshouse into a Controlled Environment Room (CER). Conditions were maintained at 22°C, 50% RH and 300 μmol m^−2^ s^−1^ at plant height for 12 h. Following this, anthers were dissected from the outer florets of the largest four or five spikelets on each tagged spike and placed directly into RNAlater® (Ambion, Thermo Fisher Scientific, Waltham, MA, USA). Each replicate was comprised of approximately 100 anthers taken equally from four individual spikes. Following collection, RNAlater was removed and anthers were frozen in liquid nitrogen and stored at −80°C until RNA extraction was performed. Three biological replicates were collected for each timepoint.

#### RNA isolation

Anthers were ground with liquid nitrogen and processed utilizing the Isolate II RNA Plant Kit (Bioline, Sydney, Australia), following the manufacturer's protocol “Purifying total RNA from plant tissue or filamentous fungi.” RNA samples were checked for concentration and purity using a NanoDrop ND-1000 (Thermo Fisher Scientific, Waltham, MA, USA). Samples were also tested via electrophoresis using a 2% agarose gel with SYBR Safe (Invitrogen, Carlsbad, CA, USA) to determine quality and degradation of RNA samples. Samples were frozen and stored at −80°C until required.

#### cDNA library preparation and sequencing

The six samples were prepared using a TruSeq Stranded mRNA LT Sample Prep Kit (Illumina, San Diego, CA, USA) following manufacturer's protocols. 1 μg of total RNA was processed for each sample, using the LS (low sample) protocol. Poly(A) mRNA was isolated from the total RNA with magnetic oligo beads. A fragmentation time of 8 min was utilized to achieve an average library size of 309 bp with a median insert length of 155 bp. Following library preparation, samples were assessed using an Agilent 2200 TapeStation (Agilent Technologies, Santa Clara, CA, USA) to determine quality, concentration, and fragment size. Samples with adapter dimers were re-purified with Agencourt AMPure XP beads (Beckman Coulter, Brea, CA, USA) in a 1.1:1 bead:sample ratio, before being re-assessed with the tapestation. Sample normalization was performed and was assessed using a BMG SPECTROstar Nano (BMG Labtech, Offenburg, Germany). 75 bp paired end read sequencing was performed on a NextSeq 500 desktop sequencer (Illumina, San Diego, CA, USA). Samples were randomized across approximately 12.5% of two lanes of the NextSeq 500. The two independent runs had quality scores of % ≥ Q30 91.6% and 89.8% respectively for the two runs.

#### Assembly and analysis

The fastQ files for all samples were demultiplexed with CASAVA v1.8 (Illumina). Sample read quality was analyzed using FastQC v0.11.4 (Andrews, 2010). Adapters and low quality reads (<Q25) were removed using Trimgalore v0.4.3 (Krueger, 2015). If a single read of a pair remained after this trimming it was excluded from the final counting and analysis. Reads were then aligned to an Ensembl wheat transcriptome (Brenchley et al., 2012) using HISAT v2.0.5 (Kim et al., 2015). Reads mapping to multiple isoforms of the same gene were included in the overall count, utilizing a custom python script. Counts for each sample were normalized to the total number of reads for each sample using DESeq2 v1.16.1 (Love et al., 2014) on the R Studio platform v1.0.143 (Team, 2015).

## Results

### A detailed staging of developing wheat anthers

**Figure 2:**
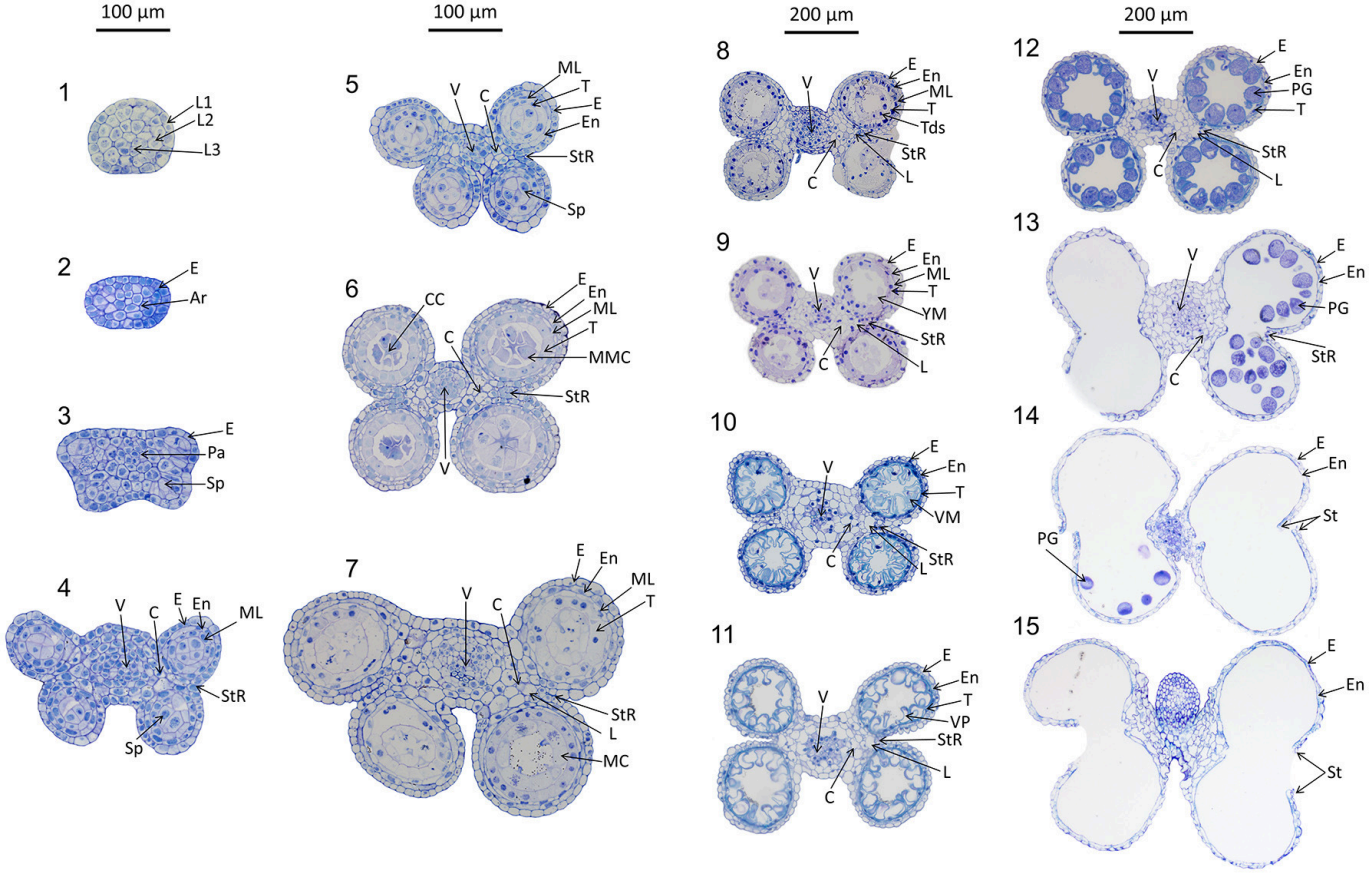
Transverse sections of wheat anthers from developmental stages 1–15. Samples were obtained from Halberd and Cranbrook cultivar wheat plants. Samples were fixed with LR White resin and stained using 1% toluidine blue. L1, 1st Cell layer; L2, 2nd Cell Layer; L3, 3rd Cell layer; Pa, Parietal Tissue; Sp, Sporogenous Tissue; E, Epidermis; En, Endothecium; ML, Middle Layer; T, Tapetum; L, Lacunae; StR, Stomium Region; MMC, Microspore Mother Cells; Tds, Tetrads; YM, Young Microspores; VM, Vacuolate Microspores; VP, Vacuolate Pollen; PG, Pollen Grains; MC, Meiotic Cells; V, Vascular Region; C, Connective Tissue; CC, Central Callose. Scale bar is 100 μm for stages 1–7 and 200 μm for stages 8–15.

Anthers develop from a mass of undifferentiated primordial cells to a complex set of tissues with varying functions. To examine the morphological changes during wheat anther development, anthers from Halberd and Cranbrook plants were dissected and transverse sectioned. The developmental pattern was divided into 15 distinct stages based on the progression of distinct morphological events. These stages are readily distinguishable in stained transverse sections under brightfield microscopy. Figure 2 contains the images of stages 1–15. The key morphological features that define each stage of development are listed in Table 1, along with a list of cell layers present at each developmental stage. Each stage has been given a descriptive name, based on nomenclature employed in the literature (Mizelle et al., 1989) and derived from the morphological characteristics present at specific stages. This nomenclature will facilitate the comparison of anther stages between species where anther stage numbering differs.

**Table 1:** Developmental stages of wheat anthers.

**Stage**	**Stage name**	**Description**	**Tissue present**	**Equivalent stage in Arabidopsis^2^**	**Equivalent stage in rice^3^**
1	Stamen primordia stage	Anther is round or oval, with epidermis clear. Inner tissue comprised of primordial cells with little or no differentiation.	Layer 1 (L1), Layer 2 (L2), Layer 3 (L3)	1	1
2	Archesporial stage	Anther becomes oval in shape. Archesporial tissue begins to form the connective tissue to separate the theca of the anther.	Epidermis (E), Archesporial tissue (Ar)	2	2
3	Sporogenous tissue stage	Four independent lobes begin to form, with parietal and sporogenous tissue present at corners of anther. Vascular region begins to form.	E, Connective tissue (C), Parietal tissue (Pa), Sporogenous tissue (Sp), Vascular region (V)	3	3
4	Lobe formation stage	Four lobes are separate and clearly defined, each containing an endothecium and middle layer. Sporogenous tissue is still present. Vascular tissue is more highly developed.	E, Endothecium (En), Middle Layer (ML), V, C	No equivalent Arabidopsis stage	4
5	Pre-callose stage^1^	Tapetal layer is present around the locules. Upper and lower locules separate at the stomium region and maintain distinct epidermal layers.	E, En, ML, V, C, Tapetum (T)	4	5
6	Central callose stage^1^	Tapetal cells grow larger as tapetum becomes complete and uniform. Microspore Mother Cells form from sporogenous tissue. Callose is visible in the center of the locules, encasing the MMCs.	E, En, ML, V, C, T, Microspore Mother Cells (MMC)	5	6
7	Meiotic stage^1^	MMCs begin to undergo meiosis as the tapetum continues to grow and vacuolate. Anther size increases and meiotic microspores move to wall of tapetum, leaving a hollow region in the center of the locules. Meiotic cells exhibit globular shape as they move toward tapetal wall.	E, En, ML, V, C, T, Meiotic Cells (MC)	6	7
8	Tetrad stage^1^	Meiotic cells break apart to form tetrads. Middle layer begins to be crushed between tapetum and outer layers.	E, En, ML, T, V, C, Tetrads (Tet)	7	8 (a and b)
9	Young microspore stage^1^	Callose wall surrounding tetrads degrades and tetrads are released as young microspores. Middle layer is barely present and tapetum is at its largest and most swollen size. Young microspores are loose within the locules.	E, En, ML, T, V, C, Young Microspores (YM)	8	9
10	Vacuolate microspore stage^1^	Microspores become vacuolate and press into tapetal wall. An exine layer begins to develop on the now vacuolate microspores. Orbicules are visible between tapetum and microspores at high magnification. Tapetum begins to reduce in size as anther grows larger.	E, En, T, V, C, Microspores (MSp)	9	10
11	Vacuolate pollen stage^1^	Microspores grow larger and more spherical, becoming multi-nucleate. Two nuclei are visible in microspores. Tapetal layer is thinner and uneven as tapetal degeneration is at its peak.	E, En, T, V, C, MSp	10	11
12	3-nucleate pollen stage^1^	Pollen grains become round and starch filled. Gametes develop within pollen grains. Tapetum is very minimal or completely gone. The anther is at its largest point.	E, En, V, C, Pollen Grains (PG), Gametes (G)	11	12
13	Bilocular stage	Pollen grains dehydrate and contract. The septal region separating the upper and lower locules degrades, causing the anther to become bilocular.	E, En, PG, Septal Region (SR), Stomium Region (StR)	12	13
14	Dehiscence	Epidermal cells at stomium region degrade, causing locules to open and the now mature pollen grains to be released.	E, En, PG, Stomium (St)	13	14
15	Senescence	Pollen continues to be released as the anther becomes brittle and degrades. Eventually the senescing anther will release from the filament and fall from the flower.	E, En, PG, St	14 (a, b, and c)	Not included in rice description

The table includes numbers and descriptive names for each stage, a description of the major morphological events occurring, a list of the cell types and morphological features present at each stage, and a comparison between the developmental stages of wheat, Arabidopsis and rice.

1 *These descriptive stage names are taken from Mizelle et al. (1989)*.

2 *Arabidopsis stages are based on Sanders et al. (1999)*.

3 *Rice stages are from Zhang and Wilson (2009)*.

Of the 15 developmental stages identified, eight have been described previously by Mizelle et al. (1989). Their study described the development of the wheat anther from the appearance of the tapetal layer (Stage 5, Pre-callose stage) to the formation of mature pollen grains and degradation of the tapetum (Stage 12, 3-nucleate pollen stage).

The early stages of anther development (stages 1–5) shown in Figure 2 involve the formation of anther shape and the cellular differentiation of the four locule cell layers. The four cell layers, namely epidermis, endothecium, middle layer and tapetum, develop during this period, giving the anther its distinctive shape with its four individual locules. By stage 6, the central callose stage, the microspore mother cells develop from the sporogenous tissue. Subsequently, meiosis occurs at stage 7. At stage 9 the tetrads are released as young microspores and the tapetum is at its largest and most active, containing significantly more mitochondria than during the earlier stages (Warmke and Lee, 1978). Stage 10, the vacuolate microspore stage, is defined by the degradation firstly of the middle layer and then the tapetum. During this time the microspores are growing and accumulating sugars and other nutrients, which are released via Ubisch bodies into the locular cavity (Chapman, 1987). At stage 11 the microspores become bi-nucleated and by stage 12 contain a vegetative nucleus as well as two generative nuclei. The tapetal layer is almost fully degraded and the pollen grains are largely starch filled at stage 12. The anther becomes bilocular at stage 13 when the septum between the upper and lower locules degenerates. At stage 14 the stomium degrades, allowing dehiscence to occur. Filament elongation occurs as the anther dehisces, with the anther eventually protruding from the floret (Kirby, 2002). Stage 15 represents the completion of the developmental program of the anther, when dehiscence occurs and pollen is released. The anther senesces, eventually detaching from the filament.

### Anther staging using morphological measurements

Predicting anther developmental stages using simple measurements facilitates the analysis of tapetal and pollen development as well as the collection of anthers at specific stages. Anthers from a range of developmental stages of Halberd, Cranbrook, Young, and Wyalkatchem cultivars were fixed and sectioned, allowing accurate determination of developmental stage. Auricle distance, spike length, spikelet length, anther length and anther width were recorded for each anther sectioned (Figure 1). Data for these measurements is shown in Supplemental Tables 2, 3. Correlations of the anther stages with the measurements were then established. Cranbrook and Halberd anthers from stages 4 to 12 were collected and auricle distances were recorded for stages 6–12. Young and Wyalkatchem anthers between stages 9 and 12 were also collected.

**Figure 3:**
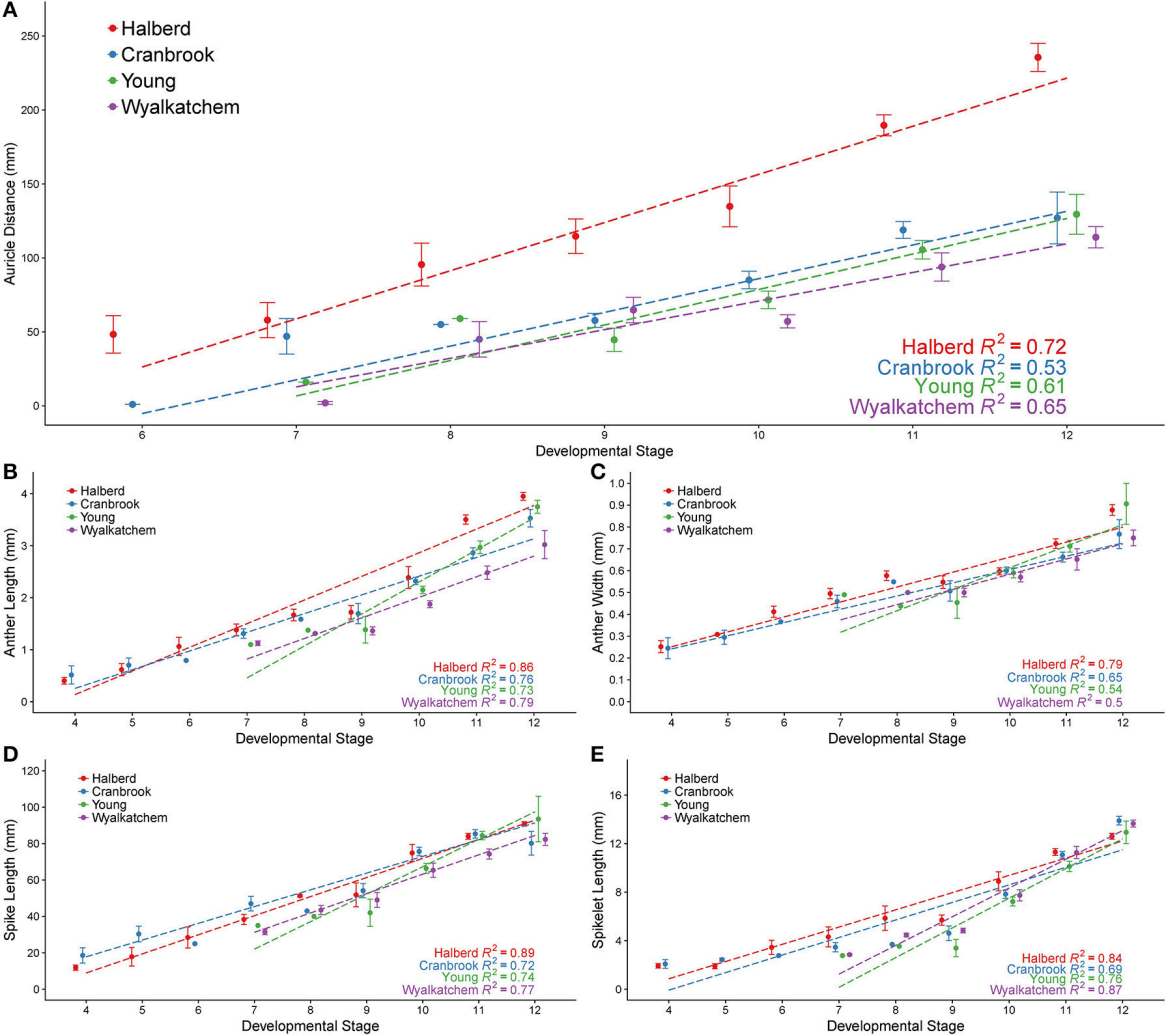
Anther developmental stages compared with five plant measurements from four wheat cultivars. Anther stages compared to auricle distance **(A)**, anther length **(B)**, anther width **(C)**, spike length **(D)**, and spikelet length **(E)**. Anthers were fixed and transverse sectioned before being staged using brightfield microscopy. For each stage, averages are shown with error bars representing standard error. Auricle distance measurements are shown for Halberd (red), Cranbrook (blue), Young (green) and Wyalkatchem (purple). Stages are numbered based on descriptions given in Table 1. Linear regression lines are shown for each cultivar in each graph.

In Halberd and Cranbrook, auricle distances gradually increased from anther stages 6 to 12 (Figure 3A). The gradual increases in auricle distances were also observed in Young and Wyalkatchem (Figure 3A). The auricle distance ranges at each anther stage often overlapped with the adjacent anther stages. The auricle distances varied greatly between cultivars, and variation increased as anther development progressed. The tall cultivar Halberd possessed longer auricle distances than the other three semi-dwarf cultivars during the various stages of anther development (Figure 3A). The shorter cultivars Cranbrook, Young, and Wyalkatchem exhibited more similar auricle distances at each anther stage (Figure 3A). Hence, auricle distance measurements may be used for anther staging of plants within a cultivar, but each cultivar must first be individually assessed.

### Anther staging using anther length and width

The anther lengths of Halberd and Cranbrook appeared to relate most closely to anther stages with the two cultivars exhibiting similar anther lengths from stages 4 to 10 (Figure 3B). However, differences between the anther lengths of the two cultivars increase at stage 11 (Figure 3B). Differences also were shown between Wyalkatchem and the other cultivars from stage 10. These results indicate that anther lengths from stages 4 to 10 are likely to be more suitable for the determination of anther stages of different cultivars, but later anther stages may be less suited to a single measurement scale, and may require individually calibrated measurements.

Anther widths of Halberd and Cranbrook increased from stages 4 to 8. However, widths from stages 8 to 10 in all four cultivars did not significantly differ (*p* < 0.05 with ANOVA test) suggesting that anther width stagnates between these important developmental stages (Figure 3C). Hence, anther length may not be a more appropriate measurement for anther developmental staging.

### Anther staging using spike length and spikelet length

Spike lengths for Halberd increased steadily from stages 4 to 12, except between stages 8 and 9 where spike growth did not increase (Figure 3D). This steady increase was not observed in Cranbrook, probably reflecting low sample numbers at several developmental stages (namely stages 6 and 8). The spikelet lengths largely exhibited similar trends to spike length, where gradual increases were seen except from stage 8 to 9. (Figure 3E). Similar increases in spike length and spikelet length were observed in Young and Wyalkatchem (Figures 3D,E) from stages 9 to 12. Consequently, both spike and spikelet length measurements may be used to stage anther development in plants within a cultivar, although spike length is a simpler and quicker measurement to collect.

### Regression statistics

Regression analyses were performed, giving the correlation coefficient (*R*^2^) between each measurement and anther stage for each cultivar (Figure 3). Positive correlations were shown for all five measurements in all four cultivars, with values ranging from 0.50 to 0.89. For each of the four cultivars, auricle distance and anther width showed the lowest *R*^2^-values of the five measurements analyzed. Spike length, spikelet length, and anther length all gave higher *R*^2^-values in all four cultivars, indicating a better correlation to anther stages than either auricle distance or anther width within a cultivar. The *R*^2^-values differed between each cultivar, with Halberd giving higher correlations to anther stage in four of the five measurements.

### Apoptosis-like PCD in the tapetum of wheat anthers

**Figure 4:**
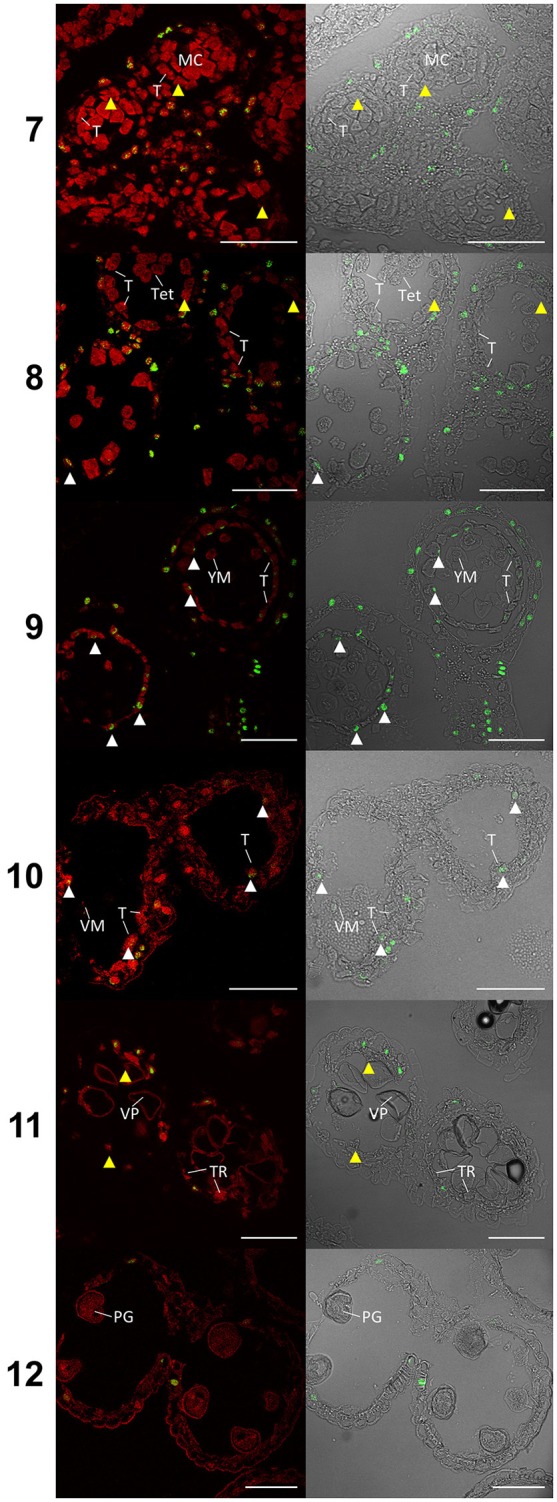
TUNEL assay of wheat cv. Halberd anthers from stage 7 to 12. Confocal microscopy of TUNEL assay in 6 μm sections of Halberd cultivar wheat anthers. Red signal is propidium iodide counter stain. Green identifies fluorescein tagged nuclei undergoing apoptosis-like Programmed Cell Death. White arrows indicate tapetal cells undergoing apoptosis-like PCD. Yellow arrows indicate tapeta where no TUNEL signal is observable. T, Tapetum; TR, Tapetal Remnant; MC, Meiotic Cell; Tet, Tetrad; YM, Young Microspore; VM, Vacuolate Microspore; VP, Vacuolate Pollen; PG, Pollen Grain. Numbers indicate anther developmental stage. Scale bars = 75 μm.

TUNEL assays detecting DNA fragmentation were performed on semi thin sections of paraffin wax embedded Halberd cultivar anthers to determine the timing of tapetal programmed cell death (PCD). Anther stages were identified in the TUNEL samples by embedding and sectioning a corresponding anther from an adjacent spikelet. This permitted the accurate identification of developmental stages of the TUNEL samples. The TUNEL signals were occasionally detected in tapetal cells at late tetrad stage (stage 8) (Figure 4) and consistently at the young microspore stage (stage 9), although TUNEL signals were observed in middle layer cells at tetrad stage. The tapetal TUNEL signals persisted to stage 10. TUNEL signals were not detected in the tapetum at the meiotic stage (stage 7). By stage 12 the tapetum has largely degraded, hence no tapetal TUNEL signal was observed at this stage. These results indicate that tapetal PCD commences between late stage 8 and early stage 9, and peaks at stage 9 of anther development.

### *MYB80* expression peaks in anthers during the tetrad and young microspore stages

**Figure 5:**
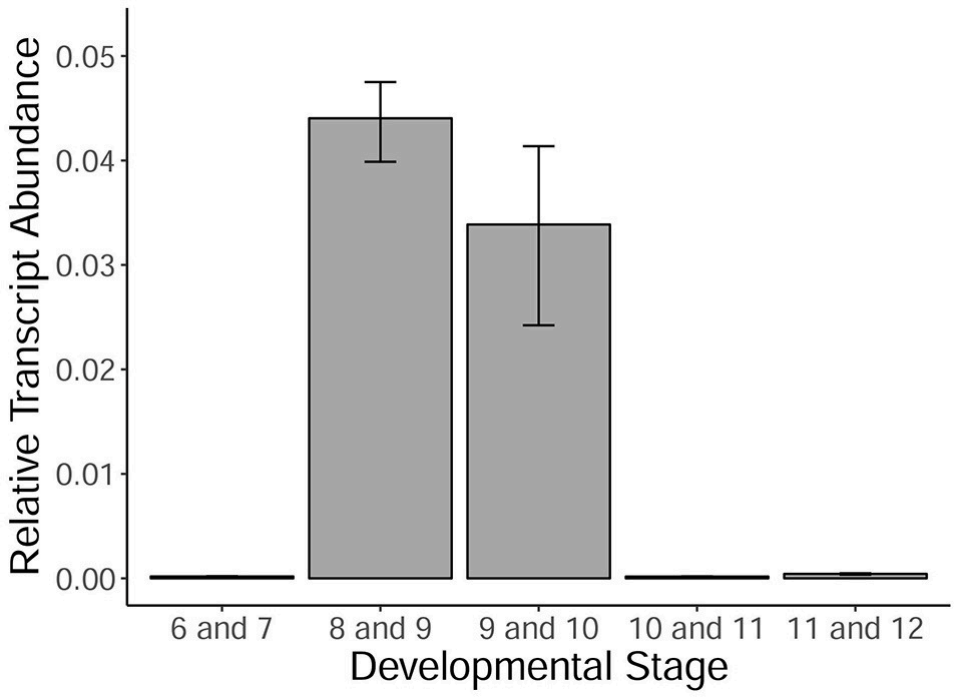
*MYB80* expression in the anthers of Halberd cultivar wheat across a variety of anther developmental stages. Each stage is the average expression of four biological replicates, each with 4 technical replicates. Expression is shown as relative transcript abundance, calculated as 2^−ΔCt^. Transcript levels are calculated relative to the average of the expression of ADP-ribosylation factor (ARF) (Ta2291) and Cell Division Control protein (CDC) (Ta54227). Error bars represent standard error.

*MYB80* is a crucial transcription factor regulating the timing of tapetal PCD, as well as callose dissolution and exine/sexine formation. Using qRT-PCR, the levels of *MYB80* expression were determined in developing Halberd cultivar anthers. A temporal expression profile was established in anthers from the central callose stage (stage 6) to the 3-nucleate pollen stage (stage 12). Expression was very low at the central callose and meiosis stages. Expression was highest in the tetrad/young microspore stage anthers and subsequently decreased (Figure 5). Minimal *MYB80* transcript expression could be detected from the vacuolate pollen stage through to the 3-nucleate pollen stage.

### Expression of tapetal regulatory gene orthologs

While the gene pathways regulating tapetal PCD and degeneration have not been fully elucidated in wheat, studies in rice and Arabidopsis have identified several important transcription factors that have been shown to regulate the development of the tapetum and the timing of tapetal PCD. Putative orthologs for six rice transcription factors, *MYB80, ETERNAL TAPETUM1* (*EAT1*), *TAPETUM DEGENERATION RETARDATION* (*TDR*), *GAMYB, TDR INTERACTING PROTEIN2* (*TIP2*) and *TAPETAL DEVELOPMENT*, and *FUNCTION1* (*TDF1*), were identified in wheat using protein sequence similarity. Excluding *EAT1*, these transcription factors also have known or putative orthologs in Arabidopsis involved in tapetal PCD regulation. Raw and normalized counts for these genes are shown in Supplemental Table 4.

**Figure 6:**
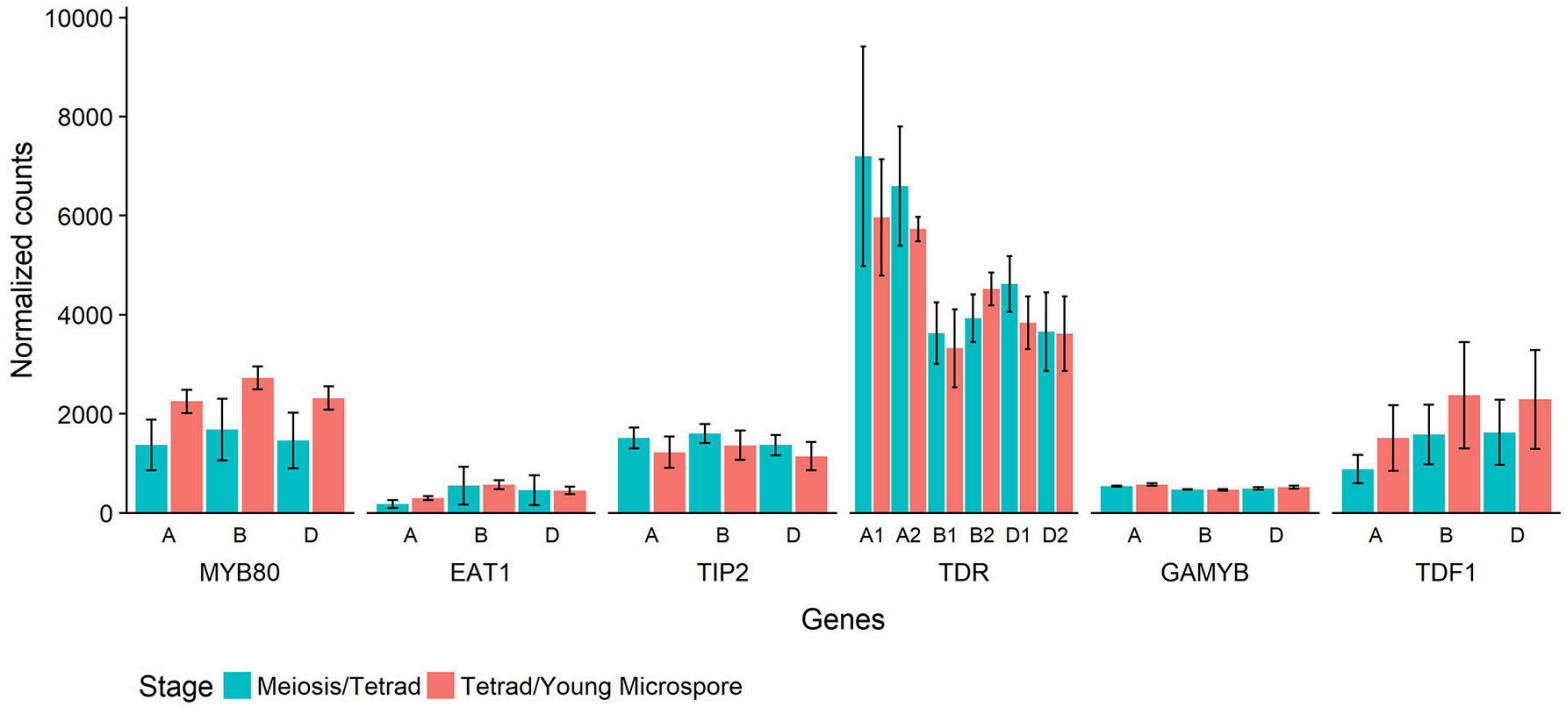
Normalized count values for putative wheat orthologs of six rice genes involved in tapetal PCD regulatory processes. Each rice gene has three wheat orthologs, or six in the case of *TDR*. The genes are identified by their rice ortholog name and which wheat genome they are present on (A, B, or D). Counts from RNAseq experiment performed on wheat cv. Halberd anther tissue, collected at meiosis/tetrad stages (blue) or tetrad/young microspore stages (red) as measured with auricle distance. Each bar represents the average of three replicates and error bars represent standard error.

In Halberd cultivar wheat, RNA was collected from anthers at two developmental timepoints, the meiosis/tetrad developmental stages and the tetrad/young microspore developmental stages. These timepoints represent the developmental stages immediately prior to and coinciding with the initiation of tapetal PCD in wheat (Figure 4). These timepoints were determined using auricle distance measurements. RNAseq analyses on these samples revealed the expression patterns of these genes in anthers at these developmental timepoints (Figure 6). For each gene, three wheat orthologs were identified, comprising genes on the A, B, and D genomes of wheat. The only exception to this was *TDR*, where a total of six orthologs were identified, two from each wheat genome. These six putative *TDR* orthologs shared at least 93% protein similarity with each other. The two *TDR* orthologs on the D wheat genome were identified as belonging to an unknown genome in the Ensembl TGACv1 genome. BLAST searches of these two genes against the IWGSCv0.4 genome confirmed their presence on the D genome.

The timepoints collected coincide with the period leading up to and including the peak expression of *MYB80*, as shown in Figure 5. These data also show expression of *MYB80* in these tissues. Increases were seen in the later stage for the three *MYB80* wheat orthologs compared to the earlier stages, although these were not statistically significant. Relative to *MYB80* values, expression of *EAT1* and *GAMYB* were lower, *TIP2* and *TDF1* count values were similar and counts for the *TDR* putative orthologs were higher. No significant differences were observed between the two timepoints for any of the genes.

## Discussion

### Anther development is similar in wheat and rice

While several stages of wheat anther development have been described (Mizelle et al., 1989), this study describes the entire anther developmental process in wheat for the first time. The anther development of wheat follows a pattern resembling that of other members of the Poaceae family, including rice (Raghavan, 1988; Zhang and Wilson, 2009) and *Brachypodium distachyon* (Sharma et al., 2015). Similarities also exist between wheat anther development and that of Arabidopsis (Sanders et al., 1999) and cotton (Xu et al., 2014) although several distinct differences were observed. In Arabidopsis and cotton, the development of the tapetal cell layer precedes the development of the four lobes. In rice and wheat, the upper and lower locules of each theca are separated at stage 4 while in Arabidopsis and cotton the two locules remain fused. As a consequence of this difference, both rice and wheat exhibit an additional developmental stage, described in Table 1 as the Lobe Formation Stage (stage 4). Thus, stage 4 of wheat and rice anther development has no equivalent stage in Arabidopsis or cotton. Rice and wheat anthers both contain lacunae, hollow spaces at the intersection of the upper and lower locules which separate the septum from the stomium (Matsui and Omasa, 2002). These lacunae are not present in Arabidopsis, cotton or *Brachypodium*. Consequently, the structures of the septum and stomium in wheat and rice anthers are quite different from those in Arabidopsis, cotton and *Brachypodium*.

Zhang and Wilson (2009) did not include a stage describing the senescence of the anther, which is equivalent to the final stage of Arabidopsis anther development (stages 14 a, b, and c). This stage is characterized by senescence of the anther and pollen release and is designated stage 15 in wheat anther development. Thus, a total of 15 developmental stages have been identified in wheat, one more than in Arabidopsis and rice. The numbering of stages varies between species such as wheat and Arabidopsis due to differences in the timing of thecae formation.

### *MYB80* expression and tapetal PCD timing

The timing of tapetal PCD is crucial for pollen development and premature or delayed tapetal PCD can result in male sterility (Kawanabe et al., 2006; Parish and Li, 2010; Parish et al., 2012). The R2R3-MYB transcription factor MYB80 regulates tapetal and pollen development including exine/sexine production (Li et al., 1999; Higginson et al., 2003; Zhang et al., 2007; Phan et al., 2011). In Arabidopsis, MYB80 also regulates callose dissolution and tetrad release via its regulation of *A6*, a putative callase resembling a β 1,3-glucanase (Zhang et al., 2007). *MYB80* is therefore crucial to the proper development of the anther. *MYB80* is conserved across a range of plant species including agriculturally important crops (Phan et al., 2012; Xu et al., 2014; Sui et al., 2015). In Arabidopsis, *MYB80* was shown to regulate the timing of tapetal PCD via the aspartic protease *UNDEAD* (Phan et al., 2011; Parish et al., 2012). Disruption of the *MYB80* gene results in premature tapetal PCD in Arabidopsis.

In Arabidopsis, *MYB80* expression commences at the meiotic stage and reaches its peak at the tetrad and young microspore stages (Li et al., 1999; Higginson et al., 2003). Tapetal PCD commences later, two stages after the peak expression, namely at Arabidopsis stage 10 (vacuolate pollen stage 11 in wheat) (Phan et al., 2011). In cotton, *MYB80* is weakly expressed at the central callose stage (stage 6) and reaches its peak expression at the tetrad stage (Xu et al., 2014). Tapetal degeneration in cotton occurs two stages after the *MYB80* peak expression at the vacuolate microspore stage (stage 9).

In rice, tapetal PCD commences at the tetrad stage (stage 8) (Li et al., 2006; Fu et al., 2014). The TUNEL signal was occasionally observed in the wheat tapetum at late tetrad stage, indicating that tapetal PCD initiation occurs at young microspore stage or late during the tetrad stage. This timing is nearly two stages earlier than in Arabidopsis anthers. *MYB80* expression was shown to be highest during the tetrad/young microspore stages in wheat (Figure 5). Hence, the initiation of tapetal PCD immediately follows the peak *MYB80* expression in wheat anthers. These results indicate that the timing of tapetal PCD are regulated differently between wheat and Arabidopsis anthers. Hence, in addition to MYB80, other factors not present in Arabidopsis may be required for the regulation of tapetal PCD in wheat.

### Predicting anther stages using auricle distance, spike length, and anther length

We examined five different plant measurements to determine their suitability for predicting anther developmental stages. There is an asynchrony in anther development between florets within a spikelet as well as between different spikelets within a single spike. Consequently, all the measurements were performed using only spikes from the main stem or largest primary tillers, and using the primary floret of the largest spikelets at the center of these spikes. Regression analyses showed a clear positive correlation between anther developmental stages and all five measurements examined. However, spike length, spikelet length, and anther length all showed higher *R*^2^-values to anther stages than auricle distance, suggesting they are better predictors of anther developmental stages than auricle distance within a cultivar.

We analyzed the frequently utilized auricle distance, as well as spike length, spikelet length, anther length, and anther width. We found that auricle distance presented the greatest variability in anther staging between cultivars (Figure 3A). Consequently, auricle distance should be measured and correlated with anther stages for each cultivar before it can be used for anther developmental staging of the cultivar. Auricle distance may be used to select tillers at the right anther developmental stages quickly and in a non-destructive manner.

Of the other four measurements, spike length proved the simplest to determine, as spikes from a young age can be measured by hand. Spike length may also have to be correlated with the anther stages for each cultivar prior to its application as it showed a degree of variation between cultivars (Figure 3D). In a field study, spike length and auricle distance would be appropriate for anther staging. For those studies which require dissection or removal of the anther, anther length measurement is the most accurate when determining anther stages. The ranges of anther lengths presented in this study (Table 2) may facilitate the prediction of anther stages in multiple cultivars up to stage 10. Beyond this developmental stage, variance in anther length between cultivars increased and cultivars may need to be individually correlated beyond this stage for accurate prediction of anther stage.

**Table 2:** Anther length ranges for anthers from stage 4 to stage 12.

**Anther stage**	**Stage name**	**Range (mm)**
4	Lobe formation	0.35–0.70
5	Pre-callose	0.50–0.85
6	Central callose	0.80–1.20
7	Meiotic	1.10–1.50
8	Tetrad	1.30–1.80
9	Young microspore	1.30–1.90
10	Vacuolate microspore	1.80–2.60
11	Vacuolate pollen	2.35–3.60
12	3-Nucleate pollen	2.75–4.00

*Lengths determined utilizing data from four wheat cultivars (Halberd, Cranbrook, Young, Wyalkatchem). Note that ranges for stages 11 and 12 are broad as cultivar specific variation is considerably greater at these developmental stages*.

Spikelet length and anther width, although both correlated with anther stage, are less suitable than spike length or anther length. Anther width often varied markedly along the length of the anther, presenting challenges in measuring width reliably. Furthermore, anther width presents a much smaller measurement than anther length, providing more difficulty in preparing anthers for measurement (Supplemental Figure 1). Spikelet length required removal of the spikelet from the spike, which occasionally resulted in spikelet destruction and made measurement difficult. A technique for measuring spikelets without requiring removal from the spike, such as a high throughput imaging technique, may make spikelet length a more viable measurement for anther stage prediction.

Anther length ranges for individual developmental stages often overlapped those of adjacent stages (Figure 3B), making it prudent to collect anthers in the middle of the size range of a specific developmental stage. In this study, anther length measurements were used to determine anther stages when analyzing *MYB80* expression in Halberd (Figure 5).

X-ray micro computed tomography has been employed to measure spike and spikelet lengths in a non-destructive manner (Tracy et al., 2017). Incorporation of this technology with developmental staging method could allow for a high-throughput plant phenotyping system to be utilized for rapid, non-destructive staging of anther development. This technique could incorporate spike length, spikelet length, and potentially anther length.

### Accurate anther stage determination

Auricle distance measurements have been used to predict the anther developmental stage for the application of abiotic stress treatments in drought (Ji et al., 2010; Onyemaobi et al., 2017) and cold (Oliver et al., 2005; Dolferus et al., 2013) in wheat, as well as heat in rice (Endo et al., 2009). Other techniques utilized to time stress application with anther development include treating plants at a specific developmental point, such as the four-leaf stage (Oshino et al., 2007) or five-leaf stage (Sakata et al., 2010) in barley. Applying stress at a specified number of days post germination (Bita et al., 2011) or using palpation to determine spike size (Dorion et al., 1996) in wheat have also been utilized. The data in this study indicate that while auricle distance can be used as a measurement for anther stage determination, measurements including spike length and anther length are more accurate and less cultivar specific, providing a more reliable method for anther stage prediction. Molecular analysis of anther development, for example, using RNAseq, requires anther staging for RNA extraction. The timing of stress applications also depends on anther developmental stages.

### Tapetal degradation regulatory network

In rice and Arabidopsis, several transcription factors have been shown to have important roles in the regulation of both the development and the degradation of the tapetal layer.

*TIP2* has been implicated in early tapetal and anther development in rice through its regulation of other genes, including *TDR* and *EAT1* (Fu et al., 2014). The expression of *TIP2* in rice anthers was shown to peak between anther stages 7 and 9. *TDR* and *EAT1* both showed their highest level of expression in anther tissue at stages 8 and 9 (Niu et al., 2013; Fu et al., 2014).

*TDR* and its Arabidopsis ortholog *AMS* regulate a number of genes involved in tapetal and pollen development and are required for correct tapetal degeneration timing (Li et al., 2006; Xu et al., 2010). *MYB80* is well described in rice and Arabidopsis and its expression was shown in this study to peak at wheat anther stages 8 and 9 (Figure 5). *EAT1* acts downstream of *TDR* and positively regulates several aspartic proteases in rice (Niu et al., 2013). *MYB80* has been shown in Arabidopsis to also positively regulate an aspartic protease (Phan et al., 2011). *TDF1* was shown to act upstream of *AMS* in Arabidopsis (Gu et al., 2014) and upstream of *TDR, EAT1* and *MYB80* in rice (Cai et al., 2015). It was found to be present in the tapetum from stages 5 to 9 in Arabidopsis (Zhu et al., 2008).

*GAMYB* has two redundant orthologs in Arabidopsis, *AtMYB33* and *AtMYB65* (Millar and Gubler, 2005). These Arabidopsis genes were shown to facilitate the timing of tapetal degradation. In rice, *GAMYB* was shown to regulate pollen formation (Kaneko et al., 2004) and GFP analysis of *GAMYB* expression in *Hordeum vulgare* indicated its expression in all four locule cell layers during early anther development, although GFP persisted in the epidermis and endothecium even after tapetal degeneration (Murray et al., 2003).

Count values for putative wheat orthologs for these six rice and Arabidopsis tapetal regulatory genes reveal their expression prior to and during the initiation of tapetal PCD in wheat anthers (Figure 6). Count values for all genes indicated gene expression at both timepoints in wheat anthers. Count values for *MYB80* match the observed expression in the qRT-PCR analysis of anther tissues at multiple developmental timepoints (Figure 5).

It is likely that the genes identified here form part of the regulatory network of anther development in wheat, including tapetal PCD initiation and degradation. This conclusion is based on the expression of the genes in anther tissue before and during the initiation of tapetal PCD, as well as their relative homology to rice and Arabidopsis genes which have been shown to regulate these processes.

## Conclusion

This study identified and described 15 stages of anther development in wheat. The morphological development of wheat and rice anthers is similar, including the timing of tapetal PCD. However, the timing and duration of tapetal PCD in wheat and Arabidopsis anthers differ significantly. Expression of *MYB80*, a gene encoding a transcription factor pivotal for pollen and anther development, was highest just prior to the commencement of tapetal PCD in wheat anthers and decreased as tapetal PCD continued. Of the five plant measurements used, spike length and auricle distance are suitable for rapid anther staging in field studies, while anther length is more suited to determining anther stages between cultivars. In the case of a new cultivar, anther length is the most suitable measurement for anther staging, without first calibrating the measurement for the specific cultivar. To use auricle distance or spike length to determine the stages of anthers, these measurements must first be calibrated with the anther stages for that specific cultivar.

## Accession number

The RNA sequences in this article have been deposited at the National Center for Biotechnology Information (NCBI) Sequence Read Archive (SRA) with the accession number SRP132420 and BioProject ID PRJNA433429.

## Author contributions

RP, SL, and RD conceived the study, RB and SI performed the experimental work, RP and SL supervised the experiments, RB drafted the manuscript, and all authors read and revised the manuscript.

### Conflict of interest statement

The authors declare that the research was conducted in the absence of any commercial or financial relationships that could be construed as a potential conflict of interest.
