# Supplementary figures and images for: Anther Morphological Development and Stage Determination in Triticum aestivum
Source: Front Plant Sci. 2018 Feb 23;9:228. doi: 10.3389/fpls.2018.00228 (PMC5829449; doi:10.3389/fpls.2018.00228)

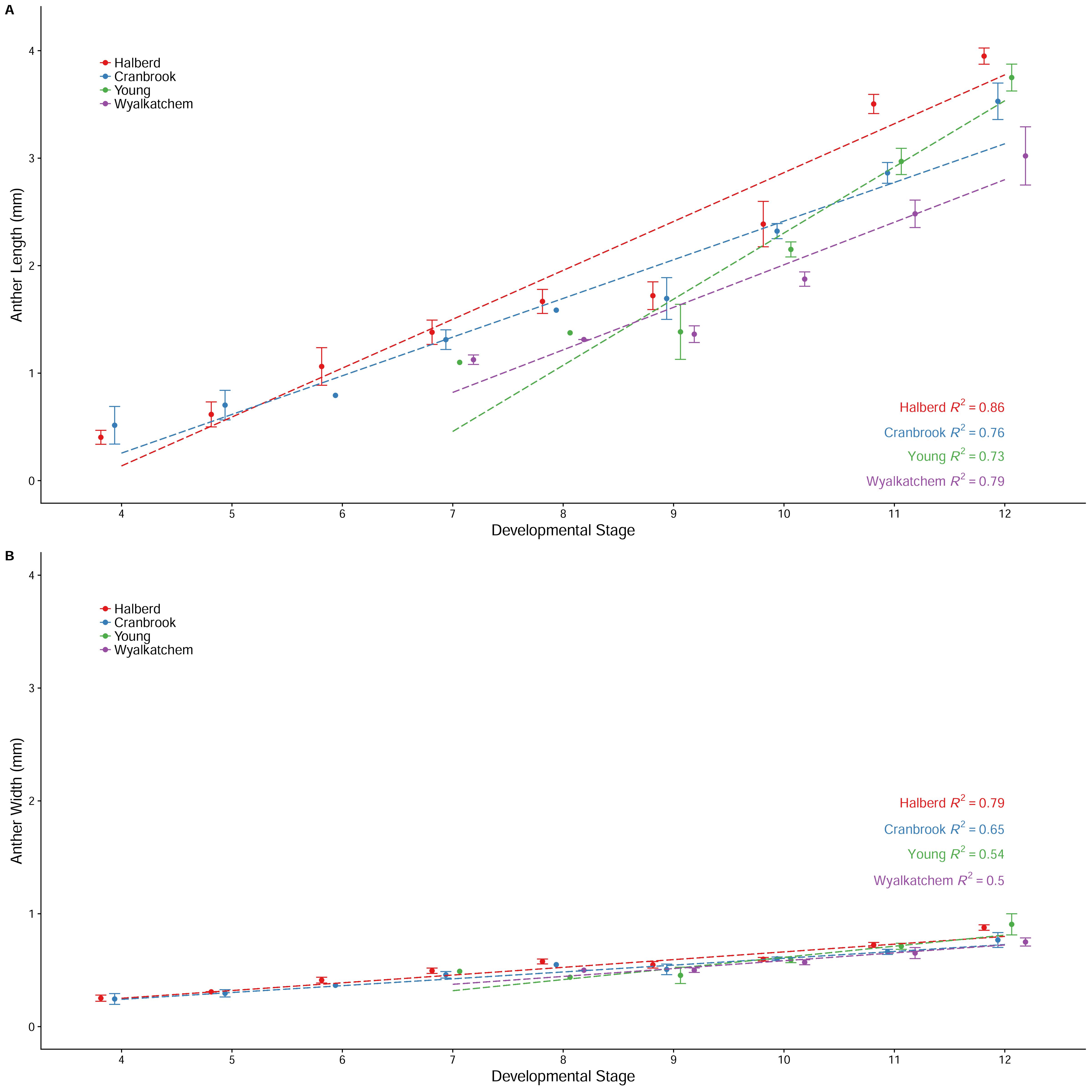

Supplement: Supplementary file 5 [file Image1.JPEG]
