# Supplementary material for: Anther Morphological Development and Stage Determination in Triticum aestivum
Source: Front Plant Sci. 2018 Feb 23;9:228. doi: 10.3389/fpls.2018.00228 (PMC5829449; doi:10.3389/fpls.2018.00228)
Supplement: Supplementary file 6 [file DataSheet1.docx]

Supplementary Material

Anther Morphological Development and Stage Determination in Triticum *aestivum*

Richard G Browne^1^, Sylvana Iacuone^1, 2^, Song F Li^1^, Rudy Dolferus^3^, Roger W Parish^1*^

*** Correspondence:** Roger W Parish, r.parish@latrobe.edu.au

# Supplementary Figures and Tables

**Supplemental Table 1:** Table containing all primer sequences used within this publication. Primers for ADP-ribosylation factor (ARF) (Ta2291) and Cell Division Control protein (CDC) (Ta54227) were obtained from Paolacci et al. (2009).

**Supplemental Table 2:** Anther development stage measurement data for each of Halberd, Cranbrook, Young, and Wyalkatchem anthers for spike length, spikelet length, anther length and anther width. Table shows average size (mm), sample size (n), and standard error for each stage. Note: Measurements for anther width of Wyalkatchem anthers at development stage 7 were not recorded.

**Supplemental Table 3:** Average size (mm) sample size (n) and standard error for each development stage measured for auricle distance measurements in each of Halberd, Cranbrook, Young, and Wyalkatchem.

**Supplemental Table 4:** Raw and normalized count values for the genes presented in Figure 6.


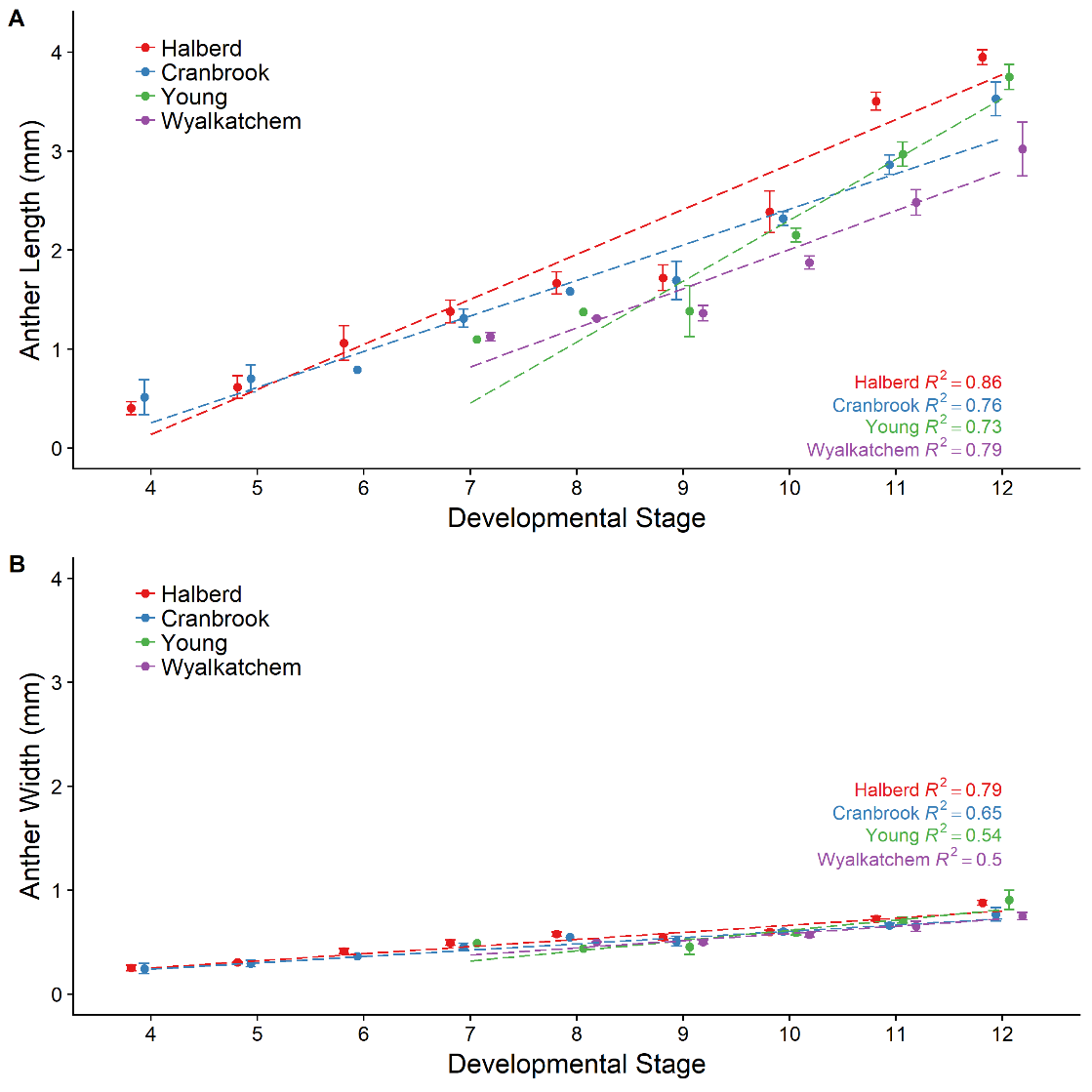


**Supplemental Figure 1:** Anther developmental stages compared with anther length **(A)** and anther width **(B)** in four wheat cultivars with identical Y-axes. Using similar methods to measure these data, it is simpler to distinguish individual stages using anther length due to the larger differences in size between individual stages. Anthers were fixed and transverse sectioned before being staged using brightfield microscopy. For each stage, averages are shown with error bars representing standard error. Auricle distance measurements are shown for Halberd (red), Cranbrook (blue), Young (green) and Wyalkatchem (purple). Stages are numbered based on descriptions given in Table 1. Linear regression lines are shown for each cultivar in each graph.
